# Towards defining muscular regions of interest from axial magnetic resonance imaging with anatomical cross-reference: a scoping review of lateral hip musculature

**DOI:** 10.1186/s12891-022-05439-x

**Published:** 2022-06-04

**Authors:** Zuzana Perraton, Peter Lawrenson, Andrea B. Mosler, James M. Elliott, Kenneth A. Weber, Natasha AMS. Flack, Jon Cornwall, Rebecca J. Crawford, Christopher Stewart, Adam I. Semciw

**Affiliations:** 1 School of Allied Health, La Trobe University, Melbourne, Australia; 2 School of Health and Rehabilitation Sciences, University of Queensland, Brisbane, Australia; 3 Department of Anatomy, School of Biomedical Sciences, The University of Otago, Dunedin, New Zealand; 4 Faculty of Medicine and Health and Northern Sydney Local Health District, The University of Sydney, The Kolling Institute, Sydney, Australia; 5 Department of Physical Therapy and Human Movement Sciences, Northwestern University, Feinberg School of Medicine, Chicago, IL USA; 6 Department of Anesthesiology, Perioperative and Pain Medicine, Stanford University, Palo Alto, CA USA; 7 University of Otago, Centre for Early Learning in Medicine, Otago Medical School, Dunedin, New Zealand; 8 Body Urbanist and Consultant, Hünenberg See, Switzerland; 9 Allied Health Research, Northern Health, Epping, VIC Australia

**Keywords:** Hip muscles, Magnetic resonance imaging, Muscle morphology, Muscle fat infiltration, Manual segmentation

## Abstract

**Background:**

Measures of hip muscle morphology and composition (e.g., muscle size and fatty infiltration) are possible with magnetic resonance imaging (MRI). Standardised protocols or guidelines do not exist for evaluation of hip muscle characteristics, hindering reliable and valid inter-study analysis. This scoping review aimed to collate and synthesise MRI methods for measuring lateral hip muscle size and fatty infiltration to inform the future development of standardised protocols.

**Methods:**

Five electronic databases (Medline, CINAHL, Embase, SportsDISCUS and AMED) were searched. Healthy or musculoskeletal pain populations that used MRI to assess lateral hip muscle size and fatty infiltration were included. Lateral hip muscles of interest included tensor fascia late (TFL), gluteus maximus, gluteus medius, and gluteus minimus. Data on MRI parameters, axial slice location, muscle size and fatty infiltrate measures were collected and analysed. Cross referencing for anatomical locations were made between MRI axial slice and E-12 anatomical plastinate sections.

**Results:**

From 2684 identified publications, 78 studies contributed data on volume (*n* = 31), cross sectional area (CSA) (*n* = 24), and fatty infiltration (*n* = 40). Heterogeneity was observed for MRI parameters and anatomical boundaries scrutinizing hip muscle size and fatty infiltration. Seven single level axial slices were identified that provided consistent CSA measurement, including three for both gluteus maximus and TFL, and four for both gluteus medius and minimus. For assessment of fatty infiltration, six axial slice locations were identified including two for TFL, and four for each of the gluteal muscles.

**Conclusions:**

Several consistent anatomical levels were identified for single axial MR slice to facilitate muscle size and fatty infiltration muscle measures at the hip, providing the basis for reliable and accurate data synthesis and improvements in the validity of future between studies analyses. This work establishes the platform for standardised methods for the MRI assessment of lateral hip musculature and will aid in the examination of musculoskeletal conditions around the hip joint. Further studies into whole muscle measures are required to further optimise methodological parameters for hip muscle assessment.

**Supplementary Information:**

The online version contains supplementary material available at 10.1186/s12891-022-05439-x.

## Background

Magnetic resonance imaging (MRI) has been used to assess skeletal muscle morphology and composition for over four decades [1–3]. Assessment of skeletal muscle with MRI can contribute to improved understanding of normal responses to physical activity and changes associated with healthy ageing, muscle injury, and pathology [1, 4]. Advancing MRI technologies, including a range of faster, higher resolution techniques continue to emerge with the aim of improving visualisation and quantification of muscle characteristics [5–7].

The use of MRI to evaluate hip muscle morphology and composition in healthy and musculoskeletal pain populations is becoming more common. Interest in hip muscle size and quality is driven by the knowledge that the muscles spanning the hip joint contribute to hip joint forces [8–10]. The capacity of a muscle to generate force has been linked to its size, including cross sectional area (CSA) and volume [11, 12]. Hip joint forces have, in turn, been associated with joint health, pain and/or other symptoms [13, 14]. How the size and quality of muscles spanning the hip joint contribute to hip joint forces is an area of particular interest [8–10].

The lateral hip muscles including the gluteus maximus, gluteus medius, gluteus minimus and the tensor fascia latae (TFL) generate forces around the hip joint for both movement and stability, particularly in single leg stance and during gait [15–18]. In people with musculoskeletal hip pain, several studies have demonstrated muscle atrophy and increased intramuscular fatty infiltration of the lateral hip muscles when compared to age-matched controls and asymptomatic contralateral limb [19–25]. As such, muscle size and fatty infiltration present as possible targets for interventions. Preliminary evidence indicates that these muscles can respond to exercises targeting the hip and other regions [26–28]. Further work assessing size and adiposity of these muscles will help to establish the most responsive type and dose of exercise to use, as well as the relationship to symptom recovery.

Recent systematic reviews have highlighted heterogeneity and inconsistencies in published MRI methods designed to assess muscle size and composition of the lateral hip muscles [7, 17, 29]. Common to all studies remains the challenges of accurately differentiating and consistently measuring the borders of individual muscles on conventional MRI which may lead to difficulties in comparing results. For the lateral hip muscles, the individual gluteal muscle borders are difficult to identify at the region between the upper border of the acetabulum and the superior tip of the greater trochanter [26, 30]. The use of high-resolution E-12 anatomical plastinates alongside MRI, may improve the ability to visualise anatomical regions by comparing and identifying key features at specific locations [5, 31, 32]. Currently, there is an urgent need for robust and reproducible MRI methods for identifying, measuring, and interpreting hip muscle images, particularly to enable comparison of results across studies and data pooling.

The primary aim of this review was to define standardised MRI methods for assessing lateral hip muscle size and fatty infiltration. A secondary aim was to provide illustrative anatomical comparisons between MRI and high-resolution E-12 anatomical plastinates using standardised locations as determined from the literature to improve visibility of muscle borders.

## Method

This review followed the PRISMA guideline extension for scoping reviews [33, 34] and was prospectively registered on the open science framework platform (https://osf.io/5nyuq/).

### Search strategy

Five electronic databases (Medline, CINAHL, Embase, SportDISCUS and AMED) were searched from inception up to Nov 1^st^ 2021. No language limits were placed. Search terms were mapped to three main concepts; (i) Magnetic resonance imaging, (ii) lateral hip muscles (i.e., TFL, gluteus maximus, gluteus medius and gluteus minimus) and, (iii) muscle morphology and composition (i.e. muscle size and fatty infiltration). Synonyms within each concept were mapped to subject headings, where possible, or searched under title, abstract and/or keywords. Results within each concept were combined with 'OR' and between concepts combined with "AND" (Additional file 1).

The search strategy was modified according to the specifications of each database. Manual citation tracking and reference checking of included articles was performed. Ahead of print lists of journals included in the study were screened for additional studies. Grey literature, such as internal reports and conference proceedings, were searched for further eligible studies.

Titles and abstracts of studies retrieved from the databases, as well as those identified from reference-checking and citation-tracking, were screened for eligibility by two reviewers (ZP and CS). Any disagreements in the eligibility of a study were discussed and a consensus reached with the aid of a third reviewer (AS). The final yield was exported into Covidence online software (www.covidence.org) for eligibility screening against inclusion and exclusion criteria.

### Inclusion/exclusion criteria

Studies with participants of any age and either healthy or musculoskeletal pain populations were included. People with cancer, neuromuscular and neurological conditions, were excluded as well as those undergoing cosmetic surgery. All MRI investigations which assessed lateral hip muscle size and/or fatty infiltrate were included. Studies were excluded if muscles were assessed as a group rather than reported individually (e.g., gluteals) and if using other imaging modalities (e.g., ultrasound) without comparison to MRI. In line with previous publications establishing regions of interest in axial images [5, 31, 35], studies using axial MRI slices for size and fatty infiltration measures were included. All published peer-reviewed studies were included; opinion pieces/editorials, systematic reviews, narrative reviews, conference abstracts and single case studies were excluded.

For our secondary aim, axial MRI images were compared to E12 anatomical plastinate sections at corresponding anatomical levels to illustrate differences, and thus identify regional morphology. The E12 anatomical plastinate sections used in this study are part of the anatomy collection, in the WD Trotter Anatomy Museum at the University of Otago. Approval to use images of the E12 plastinate sections was granted by the Department of Anatomy, University of Otago. Digital photographs were acquired of selected E12 specimens that were appropriate for the anatomical regions included in this study.

### Risk of bias (quality) assessment

The primary aim of this review was to report MRI methods rather than individual study results. As such, and in line with the PRISMA extension for scoping reviews (PRISMA-ScR) checklist [34, 36], a risk of bias assessment was not conducted.

### Data extraction

A standardised data extraction form was used to extract data relating to the individual study characteristics (study purpose, design [37], population, sample size). Countries and institution affiliations of corresponding author were recorded. Details on MRI parameters (e.g. scanner field strength, manufacturer, MRI sequence, slice selection & thickness), specific lateral hip muscles assessed, and details of size (volume and CSA) and fatty infiltration outcomes were collected by two authors (ZP and NF). Any discrepancies were discussed between authors and conflicts resolved by a third author (AS) if required.

Intraclass correlation coefficient (ICC) and the kappa coefficient (*k*) statistic are frequently used as a measure of intra- and inter-rater reliability and were collected to assess consistency of the MRI methodology between included studies [38, 39]. ICC values were interpreted as values less than 0.5 as poor reliability, 0.5 -0.75 as moderate reliability, 0.75 -0.9 as good reliability, and values greater than 0.90 as excellent reliability [38]. Kappa coefficient were interpreted as values ≤ 0.20 as none to slight, 0.21–0.40 as fair, 0.41– 0.60 as moderate, 0.61–0.80 as substantial, and 0.81–1.00 as almost perfect agreement [40]. Other measures of reliability were not collected.

### Analysis/ synthesis

Descriptive statistics were used to summarize findings across studies for MRI parameter and anatomical locations for regions of interests. Data for muscle size were grouped into volume and CSA. Fatty infiltration measures were grouped into qualitative and quantitative methods. Qualitative measures could include the Goutallier classification system [41], which grades muscle according to the relative amount of fatty tissue that is present, progressing from 0 (regular muscular tissue, no intramuscular fat) to 4 (more fat than muscle), and the Quartile classification [42] which also adopts a 5-step grading system (0%, 25%, 50%, 75% or 100%) to define the percentage of fatty tissue that is present. Quantitative measures could include various calculations incorporating fat-value pixels.

Anatomical levels for measuring CSA and fatty infiltration were collected. When a single anatomical level contained multiple anatomical features, the most easily identifiable and distinguishable anatomical feature on axial MRI slice was extracted. Axial MRI DIXON sequence images and E12 anatomical plastinate sections were cross referenced. Anatomical levels were compared on a 3D MRI image.

## Results

The initial search identified 2,684 studies, from which 1,614 duplicates were removed with a further 813 removed after title and abstract screening. An additional 176 were removed following full text screening, which resulted in 78 studies from 81 publications that met the inclusion criteria (Additional file 2 and 3).

### Trends in publication of MRI studies: 1992 to 2020

**Fig. 1:**
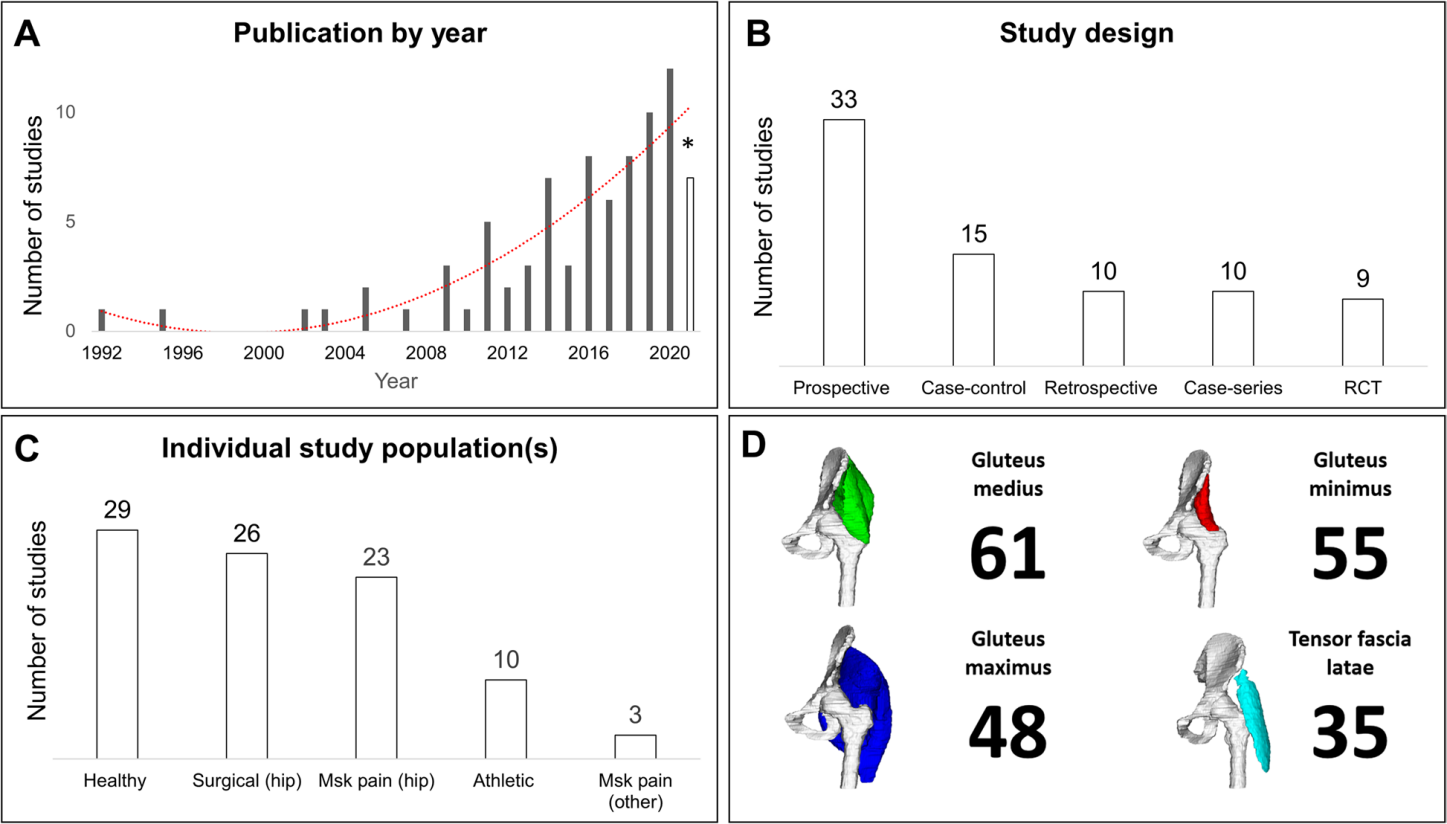
Individual study characteristics: **A** Publication year of individual studies **B** Individual study designs **C** Populations used across individual studies **D** Lateral hip muscles assessed across individual studies * Incomplete year (January to November’01); RCT Randomised control trial, Msk musculoskeletal

Frequency of publication of MRI studies has increased steadily since 1992, growing from one study [43] to 12 in 2020 [44–55] (Fig. 1A). Across the included studies, 17 countries were represented: Australia (*n* = 16), Japan (*n* = 11), Germany (*n* = 10), USA (*n* = 10), United Kingdom (*n* = 7), Switzerland (n=5), Finland (*n* = 4), France (*n* = 3), Netherlands (*n* = 3), Spain (*n* = 3), China (*n* = 2), Turkey (*n* = 2), Canada (*n* = 1), New Zealand (*n* = 1), Norway (*n* = 1), Poland (*n* = 1). Twelve institutions featured across two studies, and four institutions featured in more than two studies (Charité University Medicine, Germany *n* = 9 [42, 56–62]; La Trobe University, Australia [19, 21, 22, 63] *n* = 4, Royal National Orthopaedic Hospital, UK *n* = 3; The University of Queensland, Australia *n* = 3 [23, 24, 63]) A range of study designs were used including nine randomised controlled trials, 33 prospective cohort, 10 retrospective cohort, 15 case–control and 10 case series study designs (Fig. 1B).

### Patient and non-patient populations

Twenty-three studies across 25 publications investigated hip related musculoskeletal pain (e.g., hip osteoarthritis, lateral hip pain and intra-articular hip joint pathologies) (Fig. 1C). Three studies examined non-hip related musculoskeletal pain which included low back pain [64, 65] and patellofemoral joint osteoarthritis [66]. Twenty-nine studies, across 33 publications, used healthy comparison groups and 26 studies explored one of three surgical presentations (i.e., total hip arthroplasty, hip arthroscopy and surgical correction for hip dysplasia) (Fig. 1C). Gluteus medius was the most frequently assessed lateral hip muscle (Fig. 1D). Fifty-four studies measured muscle size and 40 studies investigated fatty infiltration (Table 1).

**Table 1:** MRI parameters for individual studies

**Citation**	**Field Strength (Tesla)**	**Axial MRI Sequence**	**Slice thickness** **(mm)** **(Gap)**	**MRI slice selection range**	**Muscle of interest**	**Size**	**Fat**	**Reliability** **(Kappa Score/ ICC)**
Ackland et al., 2019 [66]	3	● T2- FS (water excitation) ● MEDIC	1 (NR)	Sacral promontory to the inferior aspect of the pubic arch (~ 200 slices)	TFL Gmed Gmin	✓		Intra-rater ICC = 0.997 (GMin), 0.999 (Gmed), 1.00 (TFL) *5 randomly selected participants, re-evaluated, least 1 month apart*
Agten et al., 2017 [68]	1.5	● STIR & optimized inversion pulse ● T1	7 & 6 (no gap)	NR	TFL Gmax Gmed Gmin		✓	Inter-rater k = 0.548
Amabile et al., 2017 [104]	1.5	● T1 TSE	5 (no gap)	Sacrum (S1) to femoral condyles	TFL Gmax Gmed GMin	✓		NA
Arokoski et al., 2002 [82]	1.5	● T1 TSE	NR	NR	TFL Gmax Gmed Gmin	✓		NA
Belzunce et al., 2020a [52]	3	● T1 TSE ● DIXON	1.5 (0.45)	3 cm below the lesser trochanter to the top of the iliac crest	Gmax	✓		NA
Belzunce et al., 2020b[53]	3	● T1 TSE ● DIXON	1.5 & 3 (1.95 & 3.3)	Pelvis	TFL Gmax Gmed Gmin		✓	NA
Belzunce et al., 2021[91]	3	● DIXON	1.5 (1.95)	1 cm below the lesser trochanter to the top of the iliac crest	Gmax	✓	✓	NA
Berber et al., 2015 [122]	NA	● T1 TSE ● T2 TSE	NR	NR	Gmax Gmed Gmin		✓	Inter-rater k = 0.463
Bravo et al., 2013 [90]	1.5	● T1	5 (no gap)	NR	Gmax		✓	NA
Bremer et al., 2011 [93]	1.5	● T1 SE	NR	NR	TFL Gmed Gmin		✓	NA
Burian et al., 2020 [54]	3	● MEDIC	1.5 (NR)	Gluteal region	Gmax		✓	NA
Chi et al., 2015 [72]	1.5	● T1 SE ● T2- FSE fat-saturated	4–5 (1)	ASIS to the ischia (including both hips)	Gmed Gmin		✓	NA
Cowan et al., 2019 [19]	3	● T1 ● PD- with and without fat saturation	4 (NR)	Top of the iliac crest to the lesser trochanter	TFL Gmax Gmed Gmin	✓	✓	Intra-rater ICC [Size] = 0.999 *10 randomly selected muscles, re-evaluated, 6 months apart*
De Anta-Diaz et al., 2016 [94]	1.5	● T1 SE ● STIR	3–4 (NR)	NR	TFL Gmax Gmed Gmin		✓	NA
Dorado et al., 2020 [51]	1.5	● NR	8 (2)	Trunk and pelvis	Gmax Gmed Gmin	✓		NA
Ebert et al., 2019 [71]	1.5 3	● T2 fat-saturated	3.5 (NR)	NR	Gmed Gmin		✓	Inter-rater k: Ranged from 0.855 (middle portion Gmed) to 0.913 (anterior portion of the Gmin) *First 41 consecutive MRI scans were evaluated*
Emery et al., 2019 [86]	3	● PD FSE	4 (1.5)	NR	TFL	✓		Intra-rater ICC (TFL) = 1.0 (CI: 1,000, 1.000) *10 randomly selected participants, re-evaluated, 6 weeks apart*
Engelken et al., 2014 [42]	1.5	● T1 SE	4–6 (NR)	The pelvis	Gmax Gmed Gmin		✓	Intra-rater (3 observers) Quartile classification: k = 0.90, 0.90, 0.92 Goutallier classification: k = of 0.81, 0.72, 0.77
Flack et al., 2012 [73]	1.5	● T1 PD ● T1 3D-fast field echo	4 (NR)	The highest point of iliac crest superiorly to the base of the lesser trochanter	TFL Gmed Gmin	✓		Intra-rater ICC range: 0.979–0.996 (GMed), 0.886–0.995 (GMin), 0.950–0.994 (TFL)
Franettovich-Smith et al., 2017 [35]	3	● T2	5 (6)	Top of the iliac crest to the inferior gluteal fold	Gmax Gmed	✓		NA
Gerber et al., 2007 [123]	1.5	● T1 SE	8 (15)	NR	Gmax	✓		Intra-rater ICC > 0.99
Grimaldi et al., 2009 [23] (Gmax/TFL) Grimaldi et al., 2009 [24] (GMin/GMed)	1.5	● T2 FISP	6 (NR)	Iliac crest to the most distal extent of the Gmax muscle	TFL Gmax Gmed Gmin	✓		Intra-rater ICC Range: 0.870- 0.990 (TFL, Gmax) 0.985- 0.989 (Gmed, Gmin) *1 participant, all slices re-evaluated, 6 weeks apart*
Handsfield et al., 2014 [105]	3	● 2D multi-slice sequence utilising spiral gradient echo	5 (NR)	Iliac crest to the ankle joint	TFL Gmax Gmed Gmin	✓		NA
Homma et al., 2019 [107]	NA	● T1 FSE	3 (no gap)	NR	Gmax Gmed	✓		NA
Jaegers et al., 1992 [43] Jaegers et al., 1995 [124]	1.5	● SE	5 (0.5)	Iliac crest to the head of the fibula	TFL Gmax Gmed Gmin	✓		NA
Kawasaki et al., 2017 [75]	1.5	● T1 turbo spin echo	6 (NR)	NR	TFL Gmed Gmin	✓	✓	Intra-rater ICC Range [Size]: 0.750- 0.990 (TFL, Gmed, Gmin) k Range [Fat]: 0.740–0.930 (TFL, Gmed, Gmin) Inter-rater ICC [Size]: 0.990 (TFL), 0.930 (Gmed), 0.840 (Gmin) k Range [Fat]: 0.790 (TFL), 0.860 (Gmed), 0.720 (Gmin)
Kheterpal et al., 2020 [44]	1.5	● FSE PD ● T2- FSE fat-supressed	3–4 (NR)	Pelvic region	Gmed Gmin		✓	NA
Kim et al., 2014 [74]	3	● T2- FS SPAIR FSE ● T1- without FS	5 (NR)	NR	Gmax		✓	NA
Kivle et al., 2018 [67]	1.5	● T1 SE ● STIR	5 (NR)	Pelvis	Gmed Gmin	✓	✓	Intra-rater ICC [size]: 0.970 (CI: 0.91–0.99) (Gmed), 0.930 (CI: 0.84–0.97) (Gmin) Inter-rater ICC [Size]: 0.980 (CI: 0.82–0.99)(Gmed), 0.950 (CI: 0.90–0.97)(Gmin) k [Fat]: mean score = 0.23 *All participants re-evaluated, 8 weeks apart for intra-reliability* *“The interobserver reliability … were calculated on the basis of the first evaluation.”*
Kiyoshige et al., 2015 [125]	1.5	● T1	NR	NR			✓	NA
Klemt et al., 2021 [120]	1.5	● T1 TSE	4 (NR)	Pelvis	Gmax Gmed Gmin		✓	Intra-rater (3 observers) k(Quartile): 0.91, 0.89, 0.85 k(Goutallier): 0.88, 0.84, 0.81 k(Bal & Lowe): 0.83, 0.77, 0.75 Inter-rater ICC (Goutallier): range 0.84–0.91 ICC (Quartile): range 0.87–0.94 ICC (Bal and Lowe): range 0.79–0.88
Koch et al., 2021 [26]	3	● T1 VIBE‐DIXON	1.1 (NR)	Centred at the pelvis	Gmax Gmed Gmin	✓	✓	Intra-rater ICC (Size) = 0.99 (CI: 0.99–0.99) *10 participants, re-evaluated, more than 3 months apart*
Kovalak et al., 2018 [95]	1.5	● T1 TSE	3.5 (1)	NR	Gmed Gmin		✓	NA
Kubo et al., 2019 [126]	FLEXART MRT-50GP	● T1 SE	10 (no gap)	The anterior superior iliac spine to distal tibia	Gmax	✓		NA
Loureiro et al., 2018 [76]	3	● T1 2D gradient-recall acquisition in the steady state	10 (1)	Approx. 2 cm superior to the iliac crest to approx.. 2 cm inferior to the proximal tibio-fibula joint	TFL Gmax Gmed Gmin	✓		Intra-rater (for all muscles) ICC > 0.985 *All image slices for a single randomly selected participant, re-evaluated, 2 weeks apart*
Makridis et al., 2014 [127]	NA	● T1	4–5 (NR)	NR	Gmed Gmin	✓	✓	NA
Malloy et al., 2019 [25]	1.5	● T1 FSE	4 (5)	Iliac crest to the level of the knee joint	TFL Gmax Gmed Gmin	✓		Inter-rater ICC = 0.989 (CI: 0.985–0.992) *“20 MRI measurements* *reviewed by 2 raters”*
Marcon et al., 2016 [89]	3	● 3-point mDIXON	NR (no gap)	Iliac crest to ischial tuberosity	Gmed Gmin	✓	✓	Intra-rater ICC (Size) = 0.900 (CI: 0.78–0.95) *‘… for the first ten measurements”*
Mastenbrook et al., 2017 [128]	1.5	● 3D FS gradient- echo	0.82 (no gap)	Pelvis (ASIS to acetabulum)	TFL Gluteals (as a group)	✓		Intra-rater ICC(TFL) = 0.990 (CI: 0.99, 1.00) *20 participants re-evaluated, 2 weeks apart*
Masuda et al., 2003 [78]	1.5	● NR	5 (15)	ASIS to the head of the fibula	Gmax Gmed Gmin	✓		NA
Mendis et al., 2014 [77]	1.5	● T2 FISP	8 (8.8)	Iliac crest to just below the lesser trochanter of the femur	TFL	✓		Intra-rater ICC Range: 0.940- 1.000 (CI: 0.78, 0.99) *“Assessed in 10 randomly selected subjects”*
Mendis et al., 2016 [64]	1.5	● T2 FISP	7 (10.5)	Top of the iliac crest to the hip joint	Gmed Gmin	✓		Intra-rater ICC Range: 0.970- 0.990 (CI: 0.81–0.99) *“Assessed in 10 randomly selected subjects”*
Mendis et al., 2020 [45]	1.5	● T2 FISP	8 (8.8)	Iliac crest to below the lesser trochanter of the femur	Gmax Gmed Gmin	✓		Intra-rater ICC Range: 0.950–0.990 (CI: 0.90, 0.99) *“Assessed in 5 randomly selected subjects”*
Miller et al., 2020 [50]	3	● T1	5 (5)	abdomen, thigh, and shank	TFL Gmax Gmed Gmin	✓		NA
Miokovic et al., 2011 [62]	1.5	● NR	6 (0.6)	From the iliac crest to the inferior-most portion of the gluteus maximus with a second sequence overlapping the gluteus maximus and extending to the knee joint line	Gmax Gmed Gmin	✓		NA
Montefiori et al., 2020 [55]	1.5	● T1	3 (NR)	NR	TFL Gmax Gmed Gmin	✓		NA
Muller et al., 2010 [56]	1.5	● T1 TSE ● TIRM	6 (NR)	NR	Gmin		✓	NA
Muller et al., 2011a [58]	1.5	● T1 TSE ● TIRM	6 (NR)	NR	Gmed Gmin		✓	Inter-rater k Range: 0.51–0.89
Muller et al., 2011b [57]	1.5	● T1	NR	NR	Gmed		✓	Inter-rater k Range: 0.51–0.89
Niinimäki et al., 2016 [129] Niinimäki et al., 2019 [130]	1.5	● T1 VIBE	1 (no gap)	From the most proximal aspect of the femoral head to the distal-most aspect of the greater trochanter	Gmax	✓		NA
Peiris et al., 2020 [46]	3	● T1 2D FSE	10 (NR)	NR	Gmax Gmed Gmin	✓	✓	Intra-rater ICC [Size] Range: 0.780–1.000 ICC [Fat] = 0.990 *Measured twice with one week apart*
Pfirrmann et al., 2005 [69]	1.5	● STIR ● T1 SE	3–4 (NR)	NR	Gmed Gmin		✓	NA
Reito et al., 2016 [131]	1.5	● T1 FSE ● STIR	6.5 (1.8)	NR	Gmax Gmed Gmin	✓		NA
Rodrı´guez-Roiz et al., 2017 [70]	1.5	● T1	5 (NR)	NR	TFL	✓		NA
Rothwell et al., 2019 [132]	3	● T1 SE	5 (no gap)	NR	TFL Gmax Gmed Gmin	✓		Intra-rater *(for all muscles)* ICC (within session): 0.970 ± 0.030 ICC (between sessions): 0.960 ± 0.030 ICC (> 6 months): 0.91 ± 0.09 *Multiple occasions, both within- and between sessions, minimum 12 h apart and up to 6 months*
Ruckenstuhl et al., 2020 [49]	NR	● MARS	NR	NR	Gmed		✓	NA
Rykov et al., 2021 [92]	1.5	● T1 ● STIR	NR	NR	TFL Gmax Gmed Gmin		✓	NA
Sakamaki et al., 2011 [133]	1.5	● T1 SE	10 (no gap)	From the first cervical vertebra to the ankle joint	Gmax	✓		NA
Semciw et al., 2016 [63]	1.5	● T1	6 (no gap)	From above the iliac crest to just below the distal aspect of the TFL	Gmed Gmin	✓		NA
Skorupska et al., 2016 [65]	1.5	● T2	4 (no gap)	From the lumbar spine down to pelvis and upper thigh	Gmax Gmed Gmin	✓		Inter-rater ICC: > 0.900 for all muscles except (R) Gmed = 0.848
Springer et al., 2012 [59]	1.5	● T1 TSE	6 (NR)	NR	Gmed Gmin	✓		NA
Sugisaki et al., 2018 [106]	1.5	● T1 echo	7 (15)	From the first lumbar vertebra (L1) to the lateral malleolus of the fibula	TFL Gmax Gmed Gmin	✓		Intra-rater ICC = 0.999 *Repeated twice for 7 participants*
Sutter et al., 2013 [85]	1.5	● T1 ● STIR	NR	NR	TFL Gmed Gmin	✓	✓	NA
Takada et al., 2018 [80]	1.5	● T1	NR	NR	TFL Gmed	✓	✓	Inter-rater ICC [Size & Fat] Range: 0.700–0.980
Takada et al., 2021 [81]	1.5	● T1	1.5 (NR)	Lower pelvis & hips	TF Gmed Gmin	✓	✓	Intra-rater ICC [Size & Fat] Range: 0.750–0.980 Inter-rater ICC [Size & Fat] Range: 0.750–0.980
Takahashi et al., 2019 [79]	1.5	● T1	6 (4)	Trunk and hip	Gmax Gmed Gmin	✓		Intra-rater (for all muscles) ICC > 0.987
Tesch et al., 2005 [88]	1 1.5	● NR	10 (NR)	NR	Gmax	✓		NA
Thaunat et al., 2018 [96]	NA	● T1 non-FS	NR	NR	Gmed Gmin		✓	NA
Tran et al., 2021 [121]	3	● T1 2-point DIXON	4 (NR)	T11 vertebral level to the inferior-most portion of the Gmax	Gmax Gmed Gmin	✓	✓	Intra-rater ICC Range [Size]: 0.938– 0.994 (CI: 0.905–0.999) *All participants, re-evaluated, at least 7 days apart*
Unis et al., 2013 [87]	1.5	● STIR	NR	Lower pelvis and both hips	TFL	✓	✓	NA
Vasarhelyi et al., 2020 [48]	NR	● T2 ● STIR	NR	NR	TFL Gmax Gmed Gmin		✓	NA
von Roth et al., 2014 [60]	1.5	● T1 TSE	6 (NR)	NR	Gmed		✓	NA
Winkler et al., 2018 [61]	NA	● T1	5 (NR)	NR	Gmed	✓	✓	NA
Yang et al., 2021 [83]		● T2 FS PD	3.5 (NR)	Hip	TFL Gmax Gmed Gmin	✓		Intra-rater ICC (Gmax- pre-op): 0.985 (CI: 0.974–0.991) ICC (Gmax post-op): 0.934 (CI: 0.885–0.963) ICC (Gmin pre-op): 0.910 (CI: 0.862–0.953) ICC (Gmin post-op): 0.951 (CI: 0.915–0.972) *Re-evaluated, 2 months apart*
Yasuda et al., 2014 [84]	1.5	● T1 SE	10 (no gap)	From the top edge of the great trochanter to the lateral condyle of femur	Gmax	✓		NA
Yuksel et al., 2009 [134]	1.5	● T1 ● T2 FSE	NR	NR	TFL Gmax	✓		NA
Zacharias et al., 2016 [22] Zacharias et al., 2018 [21]	3	● T1 FSE	6 (no gap)	Iliac crest to distal insertion of TFL	TFL Gmax Gmed Gmin	✓	✓	Inter-rater ICC [Size] Range: 0.800–0.980 ICC [Fat] = 0.900 *5 participants, 2 observers*
Zhao et al. 2020 [47]	3	● T2	NR	NR	TFL	✓	✓	NA

*CI* 95% Confidence Interval, *FISP* true Fast Imaging with Steady State Precession, *FS* Fat Supressed, *FSE* Fast Spin Echo, *Gmax* Gluteus maximus, *Gmed* Gluteus medius, *GMin* Gluteus Minimus, *MARS* Metal Artifact Reduction Sequence, *MEDIC* Multi-echo Data Image Combination, *NA* Not Assessed, *NR* Not Reported, *PD* Proton Density, *Pre-op* Pre-operative, *Post-op* Post-operative, *SE* Spin Echo, *SPAIR* Spectral Attenuated Inversion Recovery, *STIR* Short Tau Inversion Recovery, *T1* T1 weighted, *T2* T2 weighted, *TFL* Tensor Fascia Latae, *TSE* Turbo Spin Echo, *TIRM* Turbo-inversion Recovery Magnitude, *VIBE* gradient echo Volumetric Interpolated Breath-hold

#### Measurement of muscle size and quality

Thirty-six studies reported the profession of the individual(s) interpreting MRIs and calculating size and fatty infiltration measures. The most frequently cited professionals were radiologists (31 studies) with 15 studies reporting radiologists with further training in musculoskeletal presentations. Other health professionals included orthopaedic surgeons and physiotherapists. Ten studies [44, 65, 67–74] reported years of experience for those who interpreted the MRIs, which ranged from 1 to 28 years.

ICC or kappa scores were reported in 33 studies (42%). For size measures, ICC scores reflected moderate to excellent reliability, with data ranging from 0.75 to 1.00 for intra-reliability and 0.70 to 0.99 for inter-reliability. Fatty infiltration ICC values indicated moderate to excellent reliability with scores ranging from 0.75 to 0.99 for intra-rater reliability and 0.70 to 0.99 for inter-rater reliability. However kappa coefficient scores were only performed for fatty infiltrate and demonstrated a greater variety of scores spanning from fair to almost perfect agreement among studies. Kappa scores ranged from 0.72 to 0.93 for intra-rater and 0.23 to 0.94 for inter-rater reliability (Table 2). No study reported scan to rescan reliability.

**Table 2:** Volume measurement outcomes for individual studies

Citation	Muscle segmentation: Manual/ automatic	Volume Full/partial	Software used	Equation
Ackland et al. 2019 [66]	Semi-automatic	Full (excl. fat)	Amira FEI (V 5.3.3, FEI Visualization Sciences Group)	Estimated from CSAs, multiplied by slice thickness, normalised to body weight
Amabile et al. 2017 [104]	Manual	Full	Imaging software developed by researchers’ institution	“Volume” was normalized to subject height
Belzunce et al., 2020 [52]	Automatic & Manual	Partial (excl. top & bottom extremes, & fat)	Simpleware ™ (V 2018.12, ScanIP)	Estimated from CSAs
Belzunce et al., 2021 [91]	Automatic	Full	Simpleware ™ (V 2020.6, ScanIP)	Estimated from CSAs, normalised to body weight
Cowan et al., 2019 [19]	Manual	Full	3D-DOCTOR (Able Software Corp.)	Estimated from CSAs, multiplied by slice thickness
Dorado 2020 et al., 2020 [51]	Manual	Full	Slice O’matic (V 4.3, Tomovision Inc)	Estimated from CSAs
Flack et al., 2012 [73]	Semi- automatic	Full (excl. fat)	OsiriX package (V 2.7.5)	Estimated from CSAs, multiplied by slice thickness
Franettovich Smith et al., 2017 [135]	Manual	Full	OsiriX package (V 5.7)	Estimated from CSAs, multiplied by slice thickness
Grimaldi et al., 2009a [23] Grimaldi et al., 2009b [24]	Manual	Full	Osiris package (V 4.19)	Estimated from CSAs, multiplied by slice thickness
Handsfield et al.,2014 [105]	Manual	Full	Software written in Matlab (The Math works Inc.)	Estimated from CSAs with normalised to body mass & height
Homma et al., 2019 [107]	Manual	Full	ZedHip (Lexi Co., Ltd.)	Estimated from CSAs, multiplied by slice thickness
Jaegers et al., 1992 [43] Jaegers et al., 1995[124]	Manual	Full	Obex (Cerebrum)	Estimated from CSAs
Koch et al., 2021 [26]	Manual	Partial	ITK‐Snap Software (V 3.6)	Estimated from CSAs
Kubo et al., 2019 [126]	Manual	Full	Osirix package (DICOM image analysis)	Estimated from CSAs, multiplied by slice thickness
Loureiro et al., 2018 [76]	Semi-automatic	Full	Mimics	Estimated from CSAs
Makridis et al., 2014 [127]	Manual	Full	NR	Estimated from CSAs, multiplied by slice thickness
Marcon et al., 2016 [89]	Semi-automatic	Full (excl. artifacts)	Myrian 1 (Intrasense)	Estimated from CSAs
Mastenbrook et al., 2017 [128]	Semi-automatic	Full	Analyze 11.0 (AnalyzeDirect, Inc.)	Estimated from CSAs
Miller et al., 2020 [50]	Manual	Full	DICOM software (V 2.2.0, Horos)	Estimated from CSAs, multiplied by slice thickness, normalised to body mass
Miokovic et al., 2011 [62]	Manual	Full	ImageJ (V 1.38x)	Estimated from CSAs
Montefiori et al., 2020 [55]	Semi-automatic	Full	Mimics (V 20.0, Materialise)	Estimated from CSAs
Reito et al., 2016 [131]	Manual	NR	NR	“Muscle atrophy was assessed as a decrease in volume and the appearance of fatty change relative to the contralateral, non-operated side.”
Rothwell et al., 2019 [132]	Manual	Full	OsiriX Lite (V 8.0.1, Pixmeo)	Estimated from CSAs, multiplied by slice thickness, normalised to body mass & height
Sakamaki et al., 2011 [133]	Manual	Full	NR	Estimated from CSAs, multiplied slice thickness
Semciw et al., 2016 [63]	Manual	Full	Sante DICOM Editor (Santesoft)	Estimated from CSAs, multiplied slice thickness
Skorupska et al., 2016 [65]	Manual	Full	ITK-SNAP (V 2.2.0)	Estimated from CSAs, multiplied by slice thickness
Springer et al., 2012 [59]	Manual	Full	Vitrea 2 (V 4.1.2.0)	Estimated from CSAs
Sugisaki et al., 2018 [106]	Manual	Full	OsiriX (V 2.4)	Estimated from CSAs, multiplied by slice thickness, normalised to body mass
Tran et al., 2021 [121]	Manual	Full	The MathWorks, Inc., Natick, MA, United States	Estimated from CSAs, multiplied by slice thickness
Winkler et al., 2018 [61]	Manual	Full	PACS workstation OsiriX	Estimated from CSAs
Zacharias et al., 2016 [22] Zacharias et al., 2018 [21]	Manual	Full (excl. fat)	Sante DICOM editor	Estimated from CSAs, multiplied by slice thickness

*CSA* Cross Sectional Area, *Excl.* Excluding, *GMax* Gluteus Maximus, *NR* Not Reported, *V* Version

#### MRI parameters

The MRI parameters of all studies are summarised in Table 1. Two MRI field strengths were reported, 1.5 Tesla and 3 Tesla. A wide range of MRI sequences were used across the studies, with many incorporating several sequence types, both T1- and T2-weighted, with and without fat suppression. Slice thickness ranged from 0.5 mm to 15 mm, with 16 studies (20.3%) not reporting slice thickness. Acquisition time ranged from 2 h 32 min [75] to 1 min 29 s [76].

All studies that reported patient positioning specified a supine position with legs extended and hips in neutral, except three studies [45, 62, 77] that used pillows under the knees for comfort, and two studies [44, 46] placing the hips into internal rotation.

#### Muscle size measures

Lateral hip muscle volume was measured in 31 studies and CSA was measured in 24 studies, (Tables 2 and 3). For volume measures, manual segmentation techniques were most frequently used (77.4%) compared to automated. For CSA, all studies used manual segmenting techniques.

**Table 3:** Cross sectional area measurement outcomes for individual studies

Citation	Muscles	Manual/ automatic	Single/multiple slice	Software used
Arokoski et al., 2002 [82]	TFL GMax GMed GMin	Manual	Single slice	NR
Emery et al., 2019 [86]	TFL	Manual	Multiple *CSA measured for four consecutive slices and mean was used*	DICOM
Homma et al., 2019 [107]	GMax GMed	Manual	Multiple *CSA measured at two anatomical levels*	ZedHip (Lexi Co., Ltd.)
Kawasaki et al., 2017 [75]	TFL GMed GMin	Manual	Single slice	NR
Kivle et al., 2018 [67]	GMed GMin	Manual	Single slice	PACS Sectra (V 16)
Malloy et al., 2019 [25]	TFL GMax GMed GMin	Manual	Single slice	Picture archiving and communication system workstation software
Masuda et al., 2003 [78]	GMax GMed GMin	Manual	Single slice *The maximum CSAs*	Public domain imaging software package (NIH image)
Mendis et al., 2014 [77]	TFL	Manual	Multiple *Mean CSA was measured from 3 consecutive slices*	Image J (V 1.43u)
Mendis et al., 2016 [64]	GMed GMin	Manual	Multiple *Mean CSA was measured from 3 consecutive slices*	Image J (V 1.4)
Mendis et al., 2020 [45]	GMax GMed GMin	Manual	Multiple *Mean CSA was measured from 3 consecutive slices*	Image J (V 1.43u)
Niinimäki et al., 2016 [129] Niinimäki et al., 2019 [130]	GMax	Manual	Single slice	Osirix program
Peiris et al., 2020 [46]	GMax GMed GMin	Manual	Single slice	Osirix program
Rodriguez-Roiz et al., 2017 [70]	TFL	Manual	Single slice	NR
Springer et al., 2012 [59]	GMed GMin	Manual	Single slice	Vitrea 2 (V 4.1.2.0)
Sutter et al., 2013 [85]	TFL GMax GMed GMin	Manual	Single slice	NR
Takada et al., 2018 [80]	TFL GMed	Manual	Single slice	ImageJ software (National Institute of Mental Health)
Takada et al., 2021 [81]	TFL GMed GMin	Manual	Single slice	ImageJ software (National Institute of Health, USA)
Takahashi et al., 2019 [79]	GMax GMed GMin	Manual	Single slice *Maximum CSAs*	ImageJ software (National Institute of Health)
Tesch et al., 2005 [88]	GMax	Manual	Single slice	Computerized planimetry
Unis et al., 2013 [87]	TFL	Manual	Single slice	NR
Yang et al., 2021 [83]	TFL GMax GMed GMin	Manual	Single slice	ImageJ software (National Institutes of Health)
Yasuda et al., 2014 [84]	GMax	Manual	Single slice	SliceOmatic software (Tomovision Incorporated)
Yuksel et al., 2009 [134]	TFL GMax	Manual	Single slice	NR
Zhao et al., 2020 [47]	TFL	NR	Single slice	NR

*CSA* Cross Sectional Area, *TFL* Tensor Fascia Latae, *Gmax* Gluteus maximus, *GMed* Gluteus Medius, *GMin* Gluteus Minimus, *NR* Not Reported, *V* Version

#### Volume measurement outcomes

Whole muscle volume was calculated for 28 studies (90.3%), while two [26, 52] measured partial muscle volume. To calculate volume, all studies incorporated sums of CSA estimates. Seventeen (54.8%) studies also incorporated slice thickness and five (16.1%) normalised calculations to either individual height or mass (Table 2).

#### Cross-sectional area measurement outcomes and axial anatomical slice location

Five studies calculated CSA from multiple slices either by using the mean derived from several consecutive slices or assessing CSA at two predetermined locations (Table 3). Single axial slices were chosen at a pre-determined anatomical locations for all other studies except for two studies [78, 79], which measured at the single slice with the greatest CSA for the individual muscle.

**Fig. 2:**
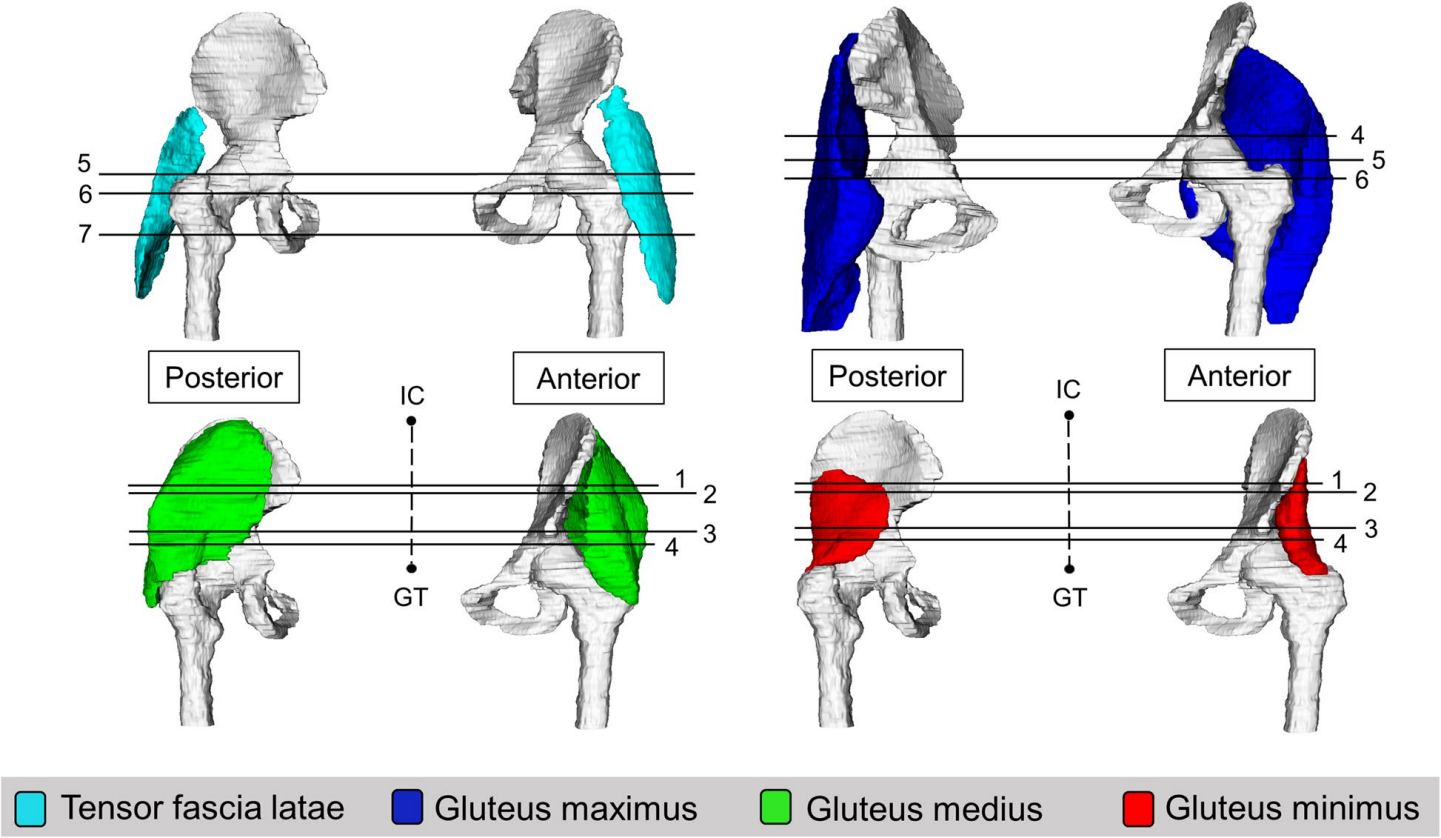
3-D representation of anatomical levels for CSA measurement; 1- Anterior superior iliac spine; 2- ½ way from iliac crest and greater trochanter; 3-Anterior inferior iliac spine; 4- Upper border of acetabulum; 5- Superior tip of greater trochanter; 6- Lower border of acetabulum; 7- Lesser trochanter; IC- Iliac crest, GT- Greater trochanter

**Fig. 3:**
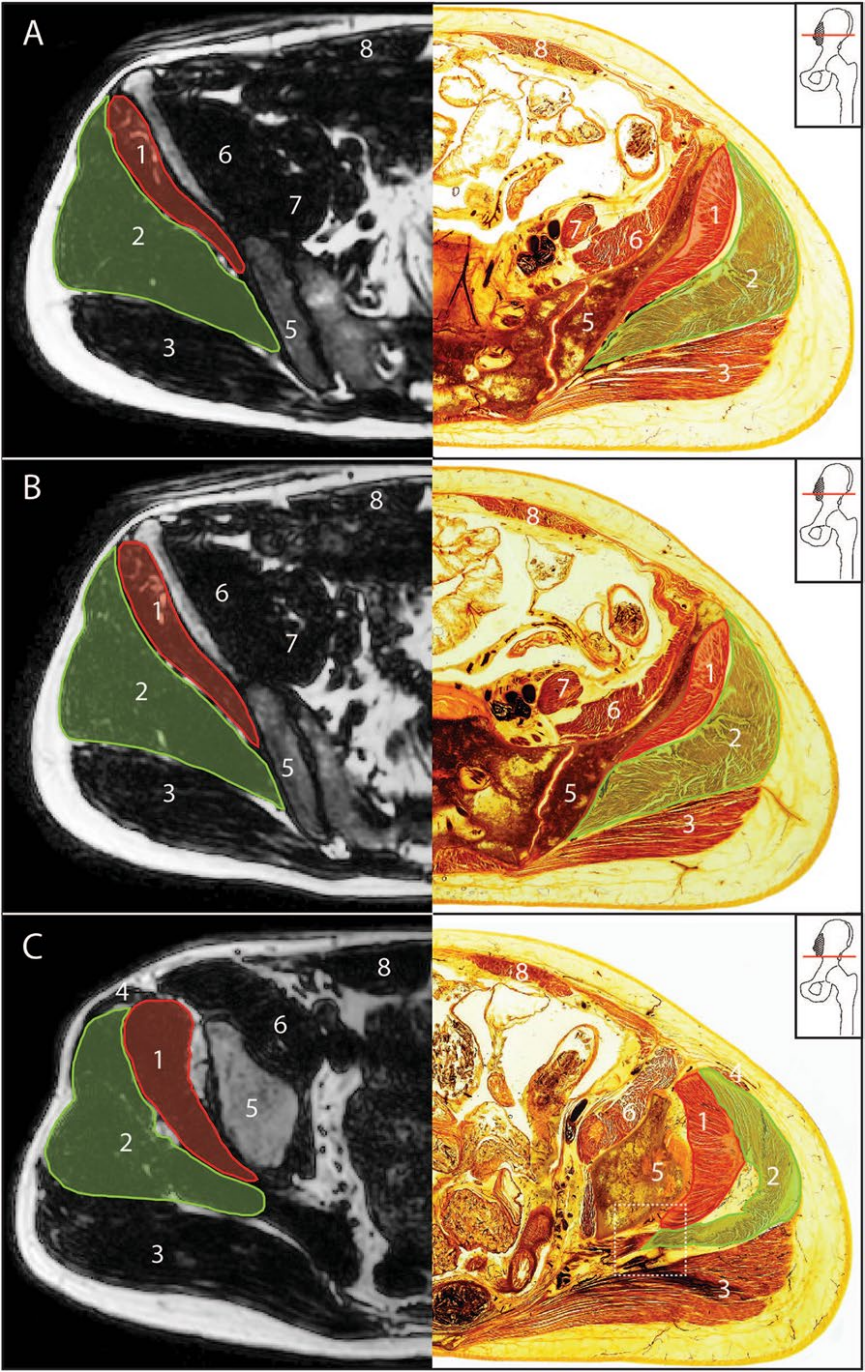
Axial DIXON sequence MRI and E-12 anatomical plastinate comparison at anatomical levels for cross sectional area measurement above the hip joint. **A** At the level of anterior superior iliac spine **B** Halfway between the iliac crest and the superior tip of the greater trochanter **C** Anterior inferior iliac spine; square dotted box surrounds enlarged morphological region of interest (Fig. 4); 1- gluteus minimus; 2- gluteus medius; 3- gluteus maximus; 4- TFL; 5- ilium; 6- iliacus; 7- psoas major; 8- rectus abdominis

**Fig. 4:**
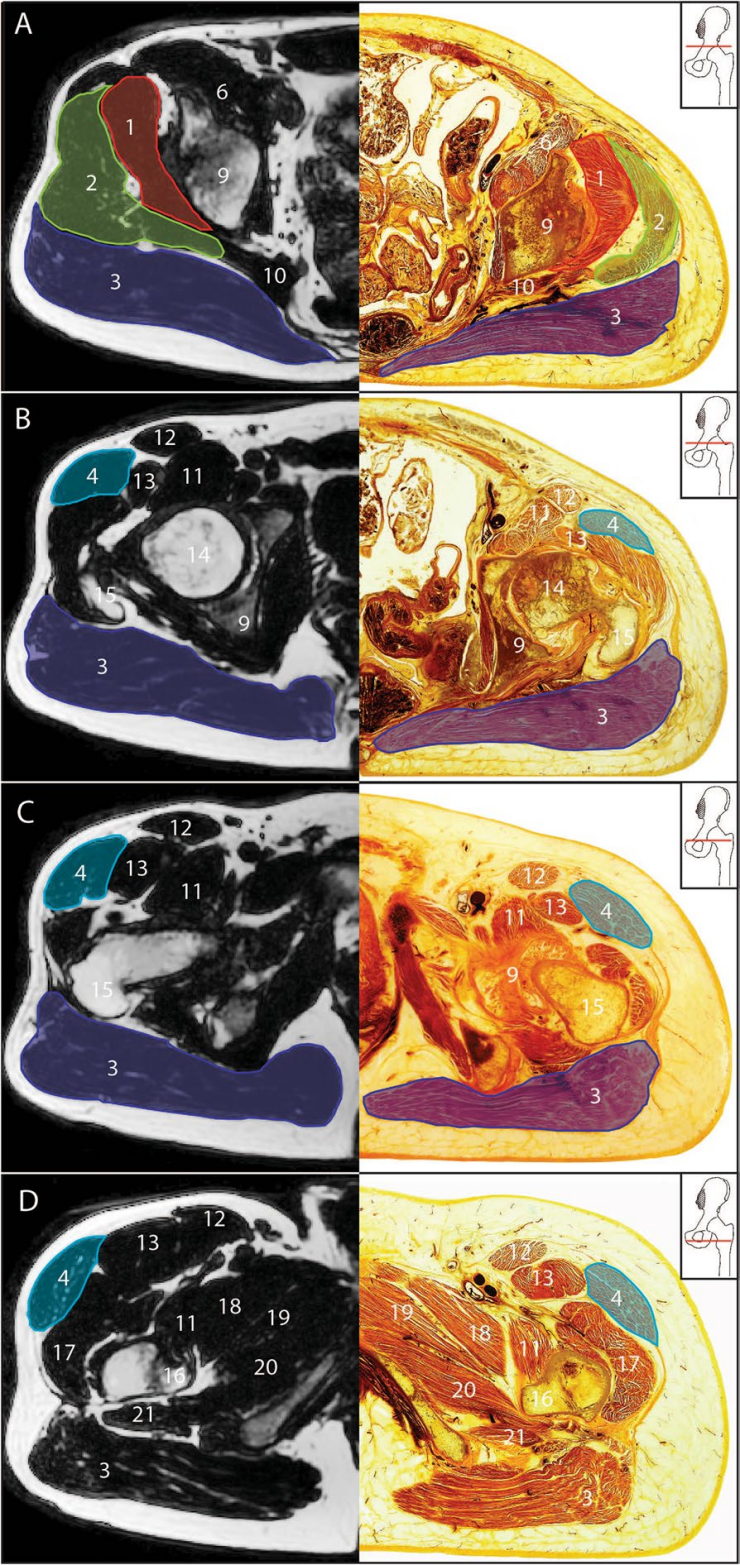
Axial DIXON sequence MRI and E-12 anatomical plastinate comparison at anatomical levels for cross sectional area measurement. **A** upper border of the acetabulum **B** superior tip of the greater trochanter **C** lower border of the acetabulum **D** lesser trochanter; 1- gluteus minimus; 2- gluteus medius; 3- gluteus maximus; 4- TFL; 6- iliacus; 9- acetabulum; 10- piriformis; 11- iliopsoas; 12- sartorius; 13-rectus femoris; 14- femoral head; 15- greater trochanter; 16- lesser trochanter; 17- vastus lateralis; 18- pectineus; 19- adductor brevis; 20- adductor magnus; 21- quadratus femoris

Seven anatomical levels were identified as locations where CSA can be measured for the lateral hip muscles (Figs. 2, 3 and 4). These include i) anterior superior iliac spine (ASIS) [59, 80, 81] ii) half way between the iliac crest and the superior tip of the greater trochanter [67] iii) anterior inferior iliac spine (AIIS) [59] iv) upper border of the acetabulum [46, 82, 83] v) superior tip of the greater trochanter [45, 70, 77, 84–87] vi) lower border of the acetabulum [25, 82, 83] and vii) lesser trochanter [57, 81].

**Fig. 5:**
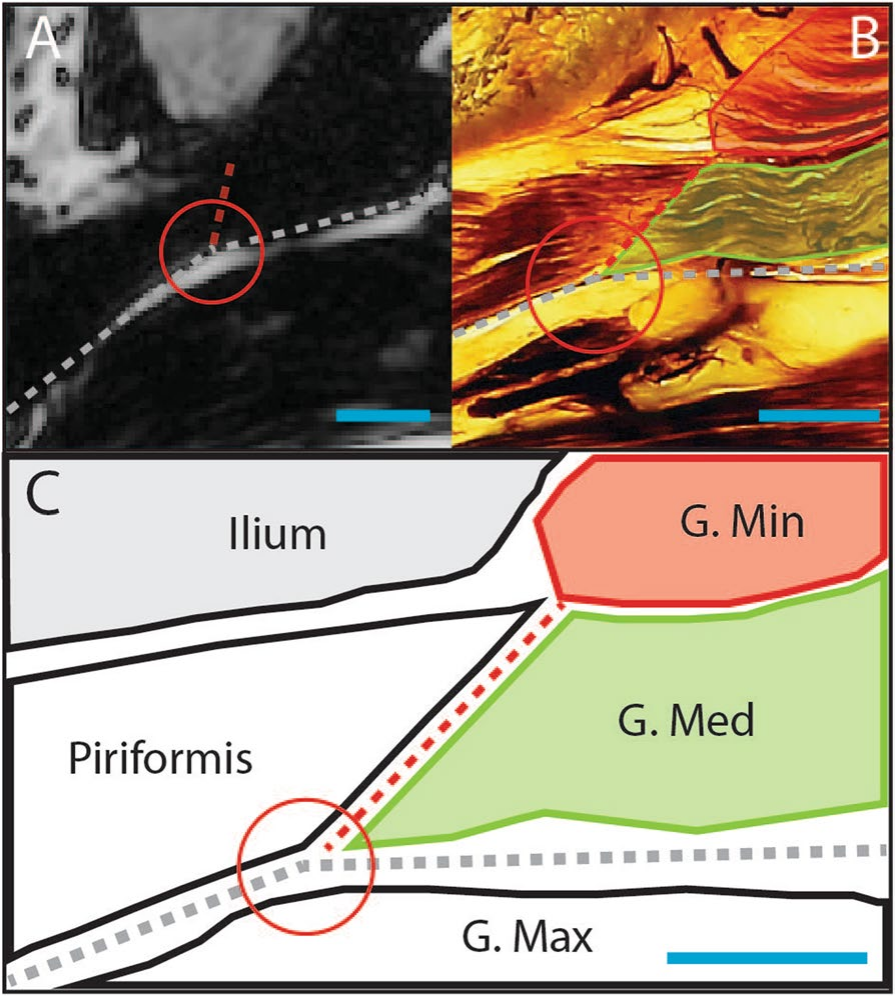
Enlarged region of interest at the level of anterior inferior iliac spine. **A** Axial DIXON sequence MRI **B** E-12 anatomical plastinate **C** Schematic illustration; round circle indicates feature of interest; Red line- gluteus minimus; Green line- gluteus medius; Dashed red line- partition between gluteus medius and piriformis; Dashed grey line- partition between gluteus maximus with both gluteus medius and piriformis; Red circle- highlights angles between partitions to help identify separation between piriformis and gluteus medius; Blue line- scale bar represents relative scale between images

When comparing MRI images to E-12 anatomical plastinates (Figs. 3 and 4), the E-12 anatomical plastinates provide better visualisation of muscle borders. At levels AIIS and the upper border of the acetabulum, the muscle borders between gluteus medius and piriformis are better visualised on the E-12 anatomical plastinates with detail of individual muscle fibre directions demarcating the individual muscles (Fig. 5). For levels at superior tip of greater trochanter and below, the TFL border is better visualised on the E-12 anatomical plastinates against neighbouring muscle borders including the gluteus medius and rectus femoris.

Some same slice locations were described in multiple ways as these levels contained multiple identifying features. For example the slice location at the level of the tip of the greater trochanter (level vi) is consistent with the level described as the centre of the femoral head [70, 85, 86], and the level where the femoral head has the greatest CSA [45], depending on slice thickness. Other slice locations were at a pre-set distance from an anatomical feature including 20 mm distal to the proximal aspect of the femoral head [88] for gluteus maximus and 15 mm from the superior margin of the acetabulum [75] for gluteus medius and minimus.

#### Intramuscular fatty infiltration measurement outcomes and axial anatomical slice location

Forty studies measured intra-muscular fatty infiltration (Table 4). Qualitative measures of fatty infiltrate were used by 30 studies with the Goutallier classification being the most frequently used. Quantification methods, using a ratio of pixel intensity from fat and water images were used by 10 studies. This technique has become more utilised over recent years.

**Table 4:** Fatty infiltration measurement outcomes for individual studies

**Citation**	**Fat Infiltration** Qualitative/Quantitative Classification system	**Divided into compartments** Yes/No
Agten et al., 2017 [68]	Qualitative G	N
Belzunce et al., 2020 [53]	Quantitative Ratio of muscle and fat value pixels	N
Belzunce et al., 2021 [91]	Quantitative Ratio of muscle and fat value pixels	N
Berber et al., 2015 [122]	Qualitative similar to G	N
Bravo et al., 2013 [90]	Quantitative Skeletal muscle lipid concentration (g /100 mL)	N
Bremer et al., 2011 [93]	Qualitative Similar to G	Y **Gmed & Gmin:** AMP
Burian et al., 2020 [54]	Quantitative Using a water-fat separation algorithm	N
Chi et al., 2015 [72]	Qualitative G	N
Cowan et al., 2019 [19]	Qualitative G	Y **Gmed & Gmin:** AP
De Anta-Diaz et al., 2016 [94]	Qualitative Grade 1: no fat or mild atrophy Grade 2: moderate or severe fatty atrophy	N
Ebert et al., 2019 [71]	Qualitative G	Y **Gmed & Gmin:** AMP
Engelken et al., 2014 [42]	Qualitative G & Q	N
Kawasaki et al., 2017 [75]	Qualitative Q	N
Kheterpal et al., 2020 [44]	Qualitative G & Q	N
Kim et al., 2014 [74]	Qualitative Similar to G	N
Kivle et al., 2018 [67]	Qualitative G	Y **Gmed & Gmin:** AMP
Klemt et al., 2021 [120]	Qualitative G & Q & Bal and Lowe classification	N
Koch et al., 2021 [26]	Quantitative Pixel intensity values from the fat only images and the water only images Muscle fat index = fat/(fat + water)	Y **Gmed:** AMP **Gmin:** AP
Kovalak et al., 2018	Qualitative G	N
Makridis et al., 2014 [127]	Qualitative G	N
Marcon et al., 2016 [89]	Quantitative Fat Signal Fraction % = 100 × fat /(water + fat)	N
Muller et al., 2010 [56]	Qualitative G	Y **Gmin:** AMP
Muller et al., 2011a[58]	Qualitative G	Y **Gmed & Gmin:** AMP
Muller et al., 2011b [57]	Qualitative G	Y **Gmed & Gmin:** AMP
Peiris et al., 2020 [46]	Qualitative Grade 0: no fat infiltration grade 1: 1–10% fat infiltration grade 2: 11–50% fat infiltration grade 3: > 50% fat infiltration	N
Pfirrmann et l., 2005 [69]	Qualitative G	Y **Gmed & Gmin:** AMP
Ruckenstuhl et al., 2020 [49]	Qualitative G	N
Rykov et al., 2021 [92]	Qualitative G	N
Sutter et al., 2013 [85]	Qualitative G	N
Takada et al., 2018 [80]	Qualitative G	N
Takada et al., 2021 [81]	Qualitative G	N
Thaunat et al., 2018 [96]	Qualitative G	Y **Gmed:** AMP
Tran et al., 2021 [121]	Quantitative Intramuscular lipid concentration	N
Unis et al., 2013 [87]	Qualitative Presence/absence	N
Vasarhelyi et al., 2020 [48]	Quantitative Muscle/fat intensity scores	N
von Roth et al., 2014 [60]	Quantitative % fat content: *“…the quotient of the number of fat-value-pixels and the number of fat-value-pixels added to the number of muscle-value pixels.”*	Y **Gmed:** AMP
Winkler et al., 2018 [61]	Quantitative % fat content = ratio of pixels of fat-value and muscle value pixels	N
Zacharias et al., 2016 [22] Zacharias et al., 2018 [21]	Qualitative G	N
Zhao et al., 2020 [47]	Qualitative G	N

*AMP* equal Anterior, Middle and Posterior thirds, *AP* equally divided into Anterior and Posterior, *FSF* Fat Single Fraction, *G* Goutallier classification, *Gmax* Gluteus maximus, *Gmed* Gluteus medius, *Gmin* Gluteus minimus, *GSF* Greater Sciatic Foramen, *GT* Greater Trochanter, *Ifat* Mean signal fat intensity, *Iwater* Mean signal water intensity, *N* No, *Q* The Quartile classification, *TFL* Tensor Fascia Latae, *Y* Yes

Gluteus medius and/or gluteus minimus were further divided into compartments in 11 studies. Gluteus medius was divided into three equal compartments (anterior, middle and posterior) by nine studies and two equal compartments (anterior, posterior) by one study. Similarly, gluteus minimus was divided into three equal compartments (anterior, middle and posterior) by seven studies and into two equal parts (anterior and posterior) by two studies. The TFL and gluteus maximus were not divided into compartments for intramuscular fatty infiltration measurement.

**Fig. 6:**
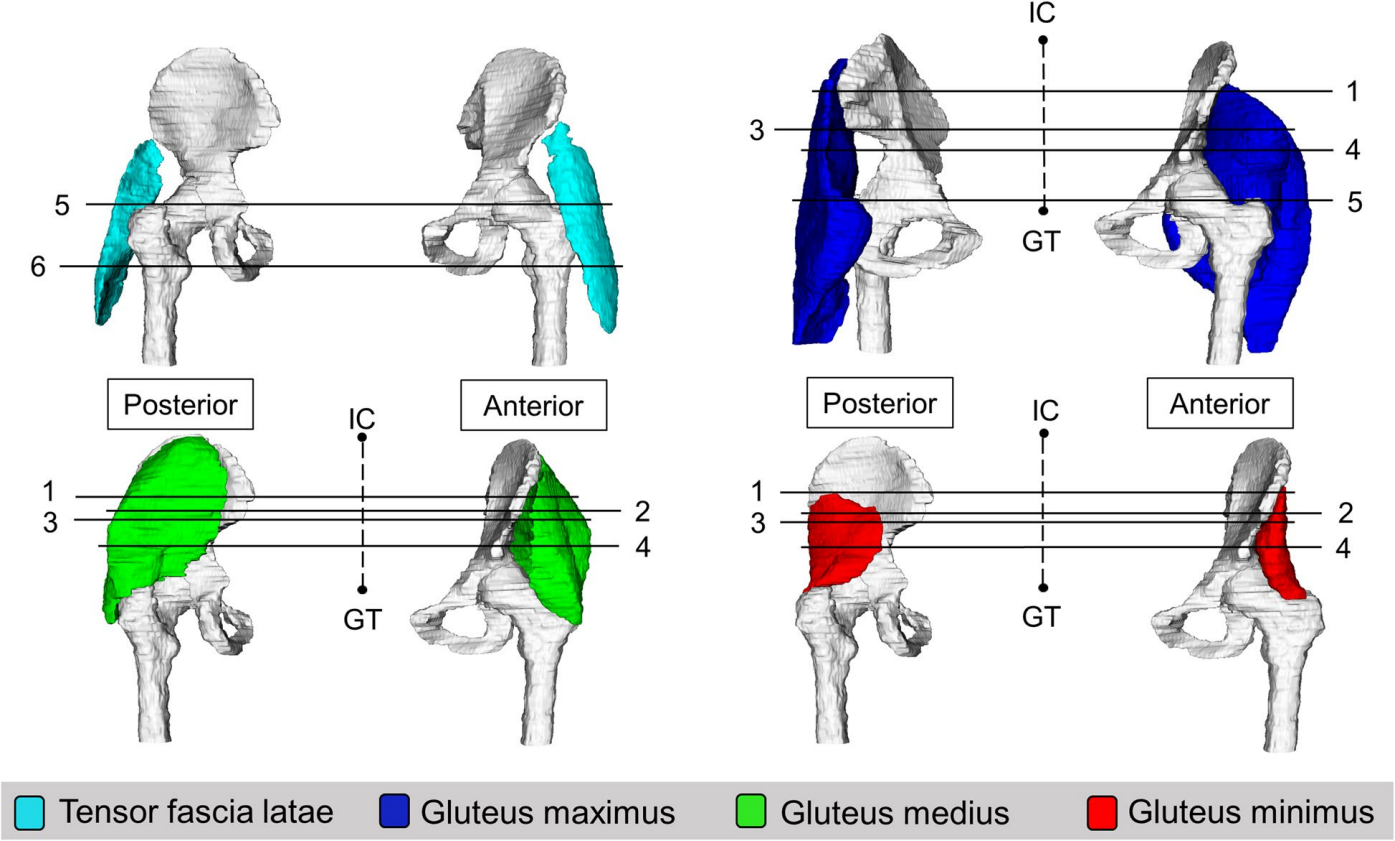
3-D representation of anatomical levels for intramuscular fatty infiltration measurement; 1- 1/3rd from iliac crest and greater trochanter; 2- Anterior superior iliac spine; 3- Greater sciatic foramen; 4- 2/3rd from iliac crest and greater trochanter; 5- GT; 6- Lesser trochanter; Aqua- TFL; Blue- Gluteus maximus; Green- Gluteus medius; Red- Gluteus minimus; IC- Iliac crest, GT- Greater trochanter

Six anatomical levels were identified as locations for fatty infiltration measurement of the lateral hip muscles (Fig. 6). Two levels were identified for TFL, four levels were identified for gluteus maximus, gluteus medius and gluteus minimus muscles. Four studies [53, 89–91] described quantitative measures of fatty infiltration for whole muscle.

### Tensor fascia latae

The two anatomical levels for TFL fatty infiltration assessment included the superior tip of the greater trochanter [85, 87] and the lesser trochanter [75, 80, 81]. The level at the greater trochanter was consistent with other anatomical features including the centre of the femoral head [85] and the fovea capitis [19, 21, 22]. The ischial tuberosity was described in one study [92] and can span multiple slices. The greatest axial CSA was described in one study [93].

### Gluteus maximus

The four levels for gluteus maximus fatty infiltration assessment are i) the distance at one third the distance from the iliac crest to the superior tip of the greater trochanter [19] ii) greater sciatic foramen (superior most part) [19, 21, 22, 42] iii) two thirds the distance from the iliac crest to the superior tip of the greater trochanter [19] iv) the superior tip of the greater trochanter [19, 94]. The level where the femoral head has a round configuration [74] and where it has the greatest circumference [19] was deemed similar to the level at the greater trochanter.

### Gluteus medius and minimus

Gluteus medius and gluteus minimus were frequently assessed individually at the same level within a study. The four levels for gluteus medius and gluteus minimus fatty infiltration assessment are i) the distance at one third the distance from the iliac crest to the superior tip of the greater trochanter [19, 67, 69, 85, 93] ii) anterior superior iliac spine [80, 81] iii) greater sciatic foramen (superior most part) [19, 21, 22, 42] and iv) two thirds the distance from the iliac crest to the superior tip of the greater trochanter[19, 56–58, 67, 69, 85, 93, 95].

Other levels described included pre-determined distances from anatomical features and included 15 mm superior to the upper margin of the acetabulum [75], three and six slices proximal to greater trochanter with slice thickness set at 6 mm [60], 30 mm proximal to greater trochanter [61]. Descriptions of levels that could span multiple axial slices included the level of the acetabulum [75, 94] and the ipsilateral sacroiliac joint [96].

#### Machine learning

Overall machine learning was incorporated in 16 (20.3%) of the studies. For size measures, eight (25.8%) studies reporting volume either used automatic or semi-automatic tracing methods while no study reporting CSA incorporated machine learning. For fatty infiltration, 10 (25.0%) studies used machine learning to identify and quantify water and fat value pixels within regions of interest.

## Discussion

This scoping review aimed to define standardised MRI methods for assessing lateral hip muscle size and fatty infiltration. When measuring size and fatty infiltration, a lack of detail and heterogeneity in reporting MRI parameters highlights the need for a consistent approach to reporting methods in future MRI research. We report seven identifiable anatomical locations for measurement of lateral hip muscle CSA and six identifiable anatomical locations for fatty infiltration at single slice measurement. We also identified new and emerging technology in machine learning for automated muscle segmentation techniques for size and fatty infiltration measures.

### MRI acquisition parameters and methodology

MRI parameters determine the quality of images that can influence the results of a study. The use of heterogenous MRI parameters, as found in this review, can complicate comparisons and future pooling of data between studies. Global, multi-centred collaborations aimed to provide MRI protocol consensus have been undertaken for other body regions and could be developed around the hip and pelvis with the aim of reducing the large variability in imaging parameters and wasted time on pilot research [97].

### Measurement

Previous studies have examined the influence of rater’s experience in reading and interpreting MRI [98, 99]. In this review, radiologists were most frequently cited professionals reading and interpreting results, with some studies specifying musculoskeletal radiologists to reflect greater experience in musculoskeletal presentations. Previous research has demonstrated MRI to be reliable for muscle size and fatty infiltration measures [6, 100]. Although the majority of studies, reporting ICC or kappa scores, stated good to excellent reliability, some studies reported fair to moderate reliability. One study [59] assessing size measures in a total hip arthroplasty population found poor reliability for measuring gluteus minimus size with analysis limited by prosthesis artifacts and poor visualisation. To overcome this limitation specific MRI techniques have since been developed for improving imaging around and near metal [101–103]. There also remains a large proportion of studies that did not report on reliability measures. This may reflect reporting bias, since poor scores would be less likely reported, and potentially inflate our estimate of reliability across the body of literature. It is recommended that future studies continue to measure and report reliability of measurement to help guide and update the development of standardised MRI methods.

### Size measures

Seven single level axial slices were identified that provided consistent CSA measurement, including three for both gluteus maximus and TFL, and four for both gluteus medius and minimus. There was no consensus on which axial slice best represents size and/or location where size changes are most likely to occur. E-12 anatomical plastinates did make visualisation of muscle borders clearer, particularly around neighbouring gluteus medius and piriformis, TFL and gluteus medius and TFL and rectus femoris. The use of E-12 anatomical plastinates in understanding and defining muscle borders at certain single level slices can aid future studies to correctly trace muscle borders and could help develop more accurate automatic, machine learning techniques.

Anatomical slice levels used in some of the included studies, where located at the very proximal or distal insertions of the target muscle which may not be representative of the muscle’s overall size. For example, the level of the anterior superior iliac spine for gluteus minimus measurement may not be the best representation for size as the muscle may not even appear at this level in some individuals. Interestingly, four studies [66, 104–106] reported size measurements from maximum CSA for individual muscles. This is supported by a recent study [107] in healthy individuals, which compared greatest CSA and volume and found a positive correlation for gluteus maximus and gluteus medius muscles. However greatest CSA may be quite different between individuals, pathologies and across studies. It is unclear at what level CSA should be calculated for the lateral hip muscles.

Compared to CSA, volume has a stronger correlation to muscle strength [12, 108], power [109], and can better reflect muscle size for the entire muscle in both healthy and musculoskeletal pain populations [7, 12]. Additionally assessing whole muscle, volume can better identify regions more susceptible to change and can inform most appropriate levels for CSA [7, 110]. For example in the thigh, after a bout of strength training in healthy individuals, muscle size changes have been observed in proximal portions of a muscle but not around distal portions [111]. Single CSA measures may therefore miss potential changes, depending on where measurements are taken. However compared to CSA, volume calculations can be more time consuming when manually derived. Supported by the results of this review, there has been an increase in interest and development of automatic calculations through machine learning. This increase will lead to greater availability of studies for future pooling of data.

### Fatty infiltration

For assessment of fatty infiltration, six axial slice locations were identified including two for TFL, four for each of the gluteal muscles. There was no consensus which axial slice best represents fatty infiltration and/or location where changes are most likely to occur. We found that 86% of studies measuring fatty infiltration used qualitative, five-point Likert scales, often at a single slice. The most frequent Likert scale used was the Goutallier classification system [41]. All studies incorporating quantitative methods for fatty infiltration studies have been published within the last 10 years reflecting it as an emerging technique.

We feel it is important to quantify muscle fat across the entire length of the muscle. This will help to identify locations where muscle fat accumulates in symptomatic groups, how it compares to asymptomatic groups, and where interventions like exercise may have the greatest effect. For example, in a study by Koch et al. [26], muscle fat was quantified on every slice from proximal to distal, and normalised to muscle length. They found that exercise had a significant effect on reducing muscle fat of gluteus minimus at the proximal portion of the muscle. If muscle fat was only measured in the distal portion, then the authors may have falsely concluded that exercise had no effect on muscle fat. In other regions of the body, Crawford and colleagues [112] have shown that the fat content at lumbar segment four (L4) best represents fatty infiltration measures that reflects the entire lumbar region in healthy participants. Further work is needed on the hip muscles to clarify if specific locations are representative of whole muscle changes.

In addition to the specific anatomical level of location, recent cadaver and electromyography studies have identified different anatomical and functional regions within the lateral hip muscles [30, 113, 114]. These compartments or regions within the individual muscle may be uniquely impacted by specific movements or muscle actions, which has relevance in musculoskeletal pathology. For example, some studies in this review divided the gluteus medius and minimus muscles into either three equal parts (anterior, middle and posterior) or two equal parts (anterior/posterior) while the gluteus maximus was divided into upper and lower portions. Investigation and understanding of muscle size and fatty infiltration within these functional regions and portions has the potential to guide future interventional studies. In spinal studies such divisions can allow for a more specific quantification to map the spatial distribution of fat content, which is increasingly showing clinical relevance as a meaningful parameter [112, 115–118].

### MRI advances

Manual tracing techniques were used for the majority of size studies but can be time consuming, involving several hours per participant. Recent advances in MRI technology include the development of automated tracing techniques through machine learning [52]. Machine learning for muscle tracing as well as for automatic fatty infiltration calculation has shown to be reliable and accurate in other regions [119]. Automated analysis incorporating machine learning is more time efficient than manual tracing, reducing analysis time from hours to seconds while still maintaining near human-level performance. However with limited valid and reliable automated methods, manual methods for labelling muscles for size and fatty infiltration are currently the gold standard [52, 91]. However, machine learning has the potential to make the analyses of larger data sets more feasible, increasing the statistical power of future research and facilitating the translation of these measures to clinical practice. Although in their infancy, automated, machine learning methods around the lateral hip muscles have shown to provide reliable data for size and the ability to quantify fatty infiltration and will aid future research [26, 48, 52, 61, 91, 120, 121].

### Limitations

This scoping review has limitations that should be considered. Firstly, this review focused on people with hip-related musculoskeletal pain and healthy populations, therefore the findings may not be generalisable to other populations such as those with neurological or muscle disease. Secondly, we acknowledge that by focusing on hip-related pain and healthy populations, additional fatty infiltration classification systems described in other populations were not included in this review. Thirdly, in addition to low reporting of reliability results, multiple studies were from single institutions which may make overall methods seem more homogeneous. Therefore, caution should be taken when generalising our findings.

Lastly, we acknowledge that a quality assessment of individual studies was not conducted. This is optional when undertaking a scoping review [34]. Reporting study quality would have a greater impact on describing the risk of bias of outcomes, rather than informing our understanding of muscle size and fat measures, which was the primary aim of this review.

## Conclusion

Whilst no consistency was found for which anatomical location(s) is(are) most appropriate and clinically meaningful to measure lateral hip muscle size and fatty infiltration, we report several identifiable anatomical levels for single axial slice muscle size and fatty infiltration. Further studies into whole muscle measures are required before strong recommendations can be made about the most suitable anatomical locations for standardised MRI single slice muscle measures and within muscle regions susceptible to change. Whilst automated machine learning technology is rapidly emerging with associated improvements in time efficiency, widespread implementation remains a challenge. Accordingly, there remains a need to optimise manual segmentation. Overall, the findings of this scoping review will assist in the future establishment of a standardised method for examination of and measurement for lateral hip musculature using MRI.

### Implications

Establishing a standardised method for MRI assessment of lateral hip muscles will contribute to greater understanding of muscle size and fatty infiltration for people with musculoskeletal conditions and the development of standardised MRI protocols. The findings of this scoping review will inform research in other clinical populations such as people with neuromuscular disease.

## Supplementary Information

**Additional file 1.** Search terms used for each main concept.

**Additional file 2.** Database search- (inception to 1^st^Nov 2021).

**Additional file 3.** Preferred Reporting Items for Systematic reviews and Meta-Analyses extension for Scoping Reviews (PRISMA-ScR) (34) flow diagram.

## Declarations

### Availability of data and materials

All the data supporting the findings are contained within the manuscript.
