# Supplementary material for: Towards defining muscular regions of interest from axial magnetic resonance imaging with anatomical cross-reference: a scoping review of lateral hip musculature
Source: BMC Musculoskelet Disord. 2022 Jun 4;23:533. doi: 10.1186/s12891-022-05439-x (PMC9166386; doi:10.1186/s12891-022-05439-x)
Supplement: Supplementary file 1 — Additional file 1. Search terms used for each main concept. [file 12891_2022_5439_MOESM1_ESM.docx]

**Additional file 1** Search terms used for each main concept

| **MRI** | **Lateral hip muscles** | **Muscle size and fatty infiltration** |
| --- | --- | --- |
| **MeSH:**  ‘Magnetic resonance imaging’  **Keywords:**  MRI OR ‘MRI scan’ OR MR OR ‘MR arthrogram’ | **MeSH:**  Gluteal Muscle*, Buttocks  **Keywords:**  ‘Hip abductor*’ OR ‘Gluteus minimus’ OR ‘gluteus medius’ OR ‘gluteus maximus’ OR gluteal* OR ‘tensor fascia lata*’ OR TFL | **Keywords:**  ‘Muscle Outcome*’ OR ‘Muscle composition’ OR ‘muscle volume’ OR ‘cross sectional area’ OR CSA OR ‘muscle size’ OR hypertrophy OR atrophy OR ‘muscle mass’ OR ‘muscle thickness’ OR ‘muscle density’ OR ‘muscle circumference’  **OR**  Fat OR adipos* OR ‘fatty infiltrat*’ OR ‘fat infiltrat*’ OR ‘intramuscular fat’ OR ‘intermuscular fat’ OR ‘muscle adipos*’ |
