# Supplementary material for: Towards defining muscular regions of interest from axial magnetic resonance imaging with anatomical cross-reference: a scoping review of lateral hip musculature
Source: BMC Musculoskelet Disord. 2022 Jun 4;23:533. doi: 10.1186/s12891-022-05439-x (PMC9166386; doi:10.1186/s12891-022-05439-x)
Supplement: Supplementary file 2 — Additional file 2. Database search- (inception to 1stNov 2021). [file 12891_2022_5439_MOESM2_ESM.docx]

**Additional file 2** Database search- (inception to 1^st^ Nov 2021)

**AMED**

| Line | Search | Results |
| --- | --- | --- |
| 1 | exp Magnetic resonance imaging/ | 1802 |
| 2 | (MRI or mri scan or MR or MR arthrogram).mp.  [mp=abstract, heading words, title] | 1782 |
| 3 | 1 or 2 | 2623 |
| 4 | exp Buttocks/ | 93 |
| 5 | (gluteal muscle ORHip abductor* or Gluteus minimus or gluteus medius or gluteus maximus or gluteal* or tensor fascia lata* or TFL).mp. [mp=abstract, heading words, title] | 572 |
| 6 | 4 or 5 | 560 |
| 7 | (Muscle Outcome* or Muscle composition or volume or cross sectional area or CSA or muscle size or  hypertrophy or atrophy or muscle mass or muscle  thickness or muscle density or muscle circumference).mp. [mp=abstract, heading words, title] | 5609 |
| 8 | (adipos* or fatty infiltrat* or fat infiltrat* or intramuscular fat or muscle adipos*).mp. [mp=abstract, heading words, title] | 619 |
| 9 | 7 or 8 | 5621 |
| 10 | 3 and 6 and 9 | 6 |

**CINAHL**

| 1 | (MH "Magnetic Resonance Imaging+") | 130,113 |
| --- | --- | --- |
| 2 | MRI OR ‘mri scan’ OR MR OR ‘MR arthrogram’ | 92,834 |
| 3 | 1 OR 2 | 155,875 |
| 4 | (MM "Buttocks") | 818 |
| 5 | (MM "Gluteal Muscles") | 112 |
| 6 | ‘Hip abductor*’ OR ‘Gluteus minimus’ OR ‘gluteus medius’ OR ‘gluteus maximu’ OR gluteal* OR ‘tensor fascia lata*’ OR TFL | 3,662 |
| 7 | 4 OR 5 OR 6 | 3,993 |
| 8 | ‘Muscle Outcome*’ OR ‘Muscle composition’ OR  volume OR ‘cross sectional area’ OR CSA OR ‘muscle size’ OR hypertrophy OR atrophy OR ‘muscle mass’ OR ‘muscle thickness’ OR ‘muscle density’ OR  ‘muscle circumference’ | 168,980 |
| 9 | adipos* OR ‘fatty infiltrat*’ OR ‘fat infiltrat*’ OR  ‘intramuscular fat’ OR ‘muscle adipos*’ | 32,752 |
| 10 | 8 OR 9 | 198,304 |
| 11 | 3 AND 7 AND 10 | 105 |

**Embase**

| 1 | exp nuclear magnetic resonance imaging/ | 1067132 |
| --- | --- | --- |
| 2 | (Magnetic resonance imaging or MRI or mri scan or MR or MR arthrogram).mp. [mp=title, abstract, heading word, drug trade name, original title, device manufacturer, drug manufacturer, device trade name, keyword, floating subheading word, candidate term word] | 1144740 |
| 3 | 1 or 2 | 1067132 |
| 4 | exp buttock/ | 9246 |
| 5 | exp gluteus muscle/ | 5948 |
| 6 | (Hip abductor* or Gluteus minimus or gluteus medius or gluteus maximus or gluteal* or tensor fascia lata* or TFL).mp. [mp=title, abstract, heading word, drug trade name, original title, device manufacturer, drug  manufacturer, device trade name, keyword, floating  subheading word, candidate term word] | 17918 |
| 7 | 4 or 5 or 6 | 23284 |
| 8 | (Muscle Outcome* or Muscle composition or volume or cross sectional area or CSA or muscle size or  hypertrophy or atrophy or muscle mass or muscle  thickness or muscle density or muscle  circumference).mp. [mp=title, abstract, heading word, drug trade name, original title, device manufacturer, drug manufacturer, device trade name, keyword, floating subheading word, candidate term word] | 1783471 |
| 9 | (adipos* or fatty infiltrat* or fat infiltrat* or intramuscular fat or muscle adipos*).mp. [mp=title, abstract, heading word, drug trade name, original title, device manufacturer, drug manufacturer, device trade name, keyword, floating subheading word, candidate term word] | 214977 |
| 10 | 8 or 9 | 1787558 |
| 11 | 3 and 7 and 10 | 1487 |

**Medline**

| 1 | exp Magnetic Resonance Imaging/ | 492867 |
| --- | --- | --- |
| 2 | (MRI or mri scan or MR or MR arthrogram).mp. [mp=title, abstract] | 397812 |
| 3 | 1 or 2 | 594852 |
| 4 | exp Buttocks/ | 6122 |
| 5 | (gluteal muscle or Hip abductor* or Gluteus minimus or gluteus medius or gluteus maximus or gluteal* or tensor fascia lata* or TFL).mp. [mp=title, abstract] | 11318 |
| 6 | 4 or 5 | 13656 |
| 7 | (Muscle Outcome* or Muscle composition or volume or cross sectional area or CSA or muscle size or  hypertrophy or atrophy or muscle mass or muscle  thickness or muscle density or muscle  circumference).mp. [mp=title, abstract] | 979617 |
| 8 | (adipos* or fatty infiltrat* or fat infiltrat* or intramuscular fat or muscle adipos*).mp. [mp=title, abstract] | 173234 |
| 9 | 7 or 8 | 1039362 |
| 10 | 3 and 6 and 9 | 1038 |

**SPORTSDiscus**

| 1 | MRI OR ‘mri scan’ OR MR OR ‘MR arthrogram’  OR 'magnetic resonance imaging' | 15,502 |
| --- | --- | --- |
| 2 | buttock* OR ‘Hip abductor*’ OR ‘Gluteus minimus’ OR ‘gluteus medius’ OR ‘gluteus maximus’ OR gluteal* OR ‘tensor fascia lata*’ OR TFL | 3,022 |
| 3 | ‘Muscle Outcome*’ OR ‘Muscle composition’ OR  volume OR ‘cross sectional area’ OR CSA OR ‘muscle size’ OR hypertrophy OR atrophy OR ‘muscle mass’ OR ‘muscle thickness’ OR ‘muscle density’ OR  ‘muscle circumference’ | 49,259 |
| 4 | adipos* OR ‘fatty infiltrat*’ OR ‘fat infiltrat*’ OR  ‘intramuscular fat’ OR ‘muscle adipos*’ | 9,234 |
| 5 | 3 OR 4 | 57,348 |
| 6 | 1 AND 2 AND 5 | 56 |
