# Supplementary material for: Towards defining muscular regions of interest from axial magnetic resonance imaging with anatomical cross-reference: a scoping review of lateral hip musculature
Source: BMC Musculoskelet Disord. 2022 Jun 4;23:533. doi: 10.1186/s12891-022-05439-x (PMC9166386; doi:10.1186/s12891-022-05439-x)

Additional file 3: Preferred Reporting Items for Systematic reviews and Meta-Analyses extension for Scoping Reviews (PRISMA-ScR) (34) flow diagram.

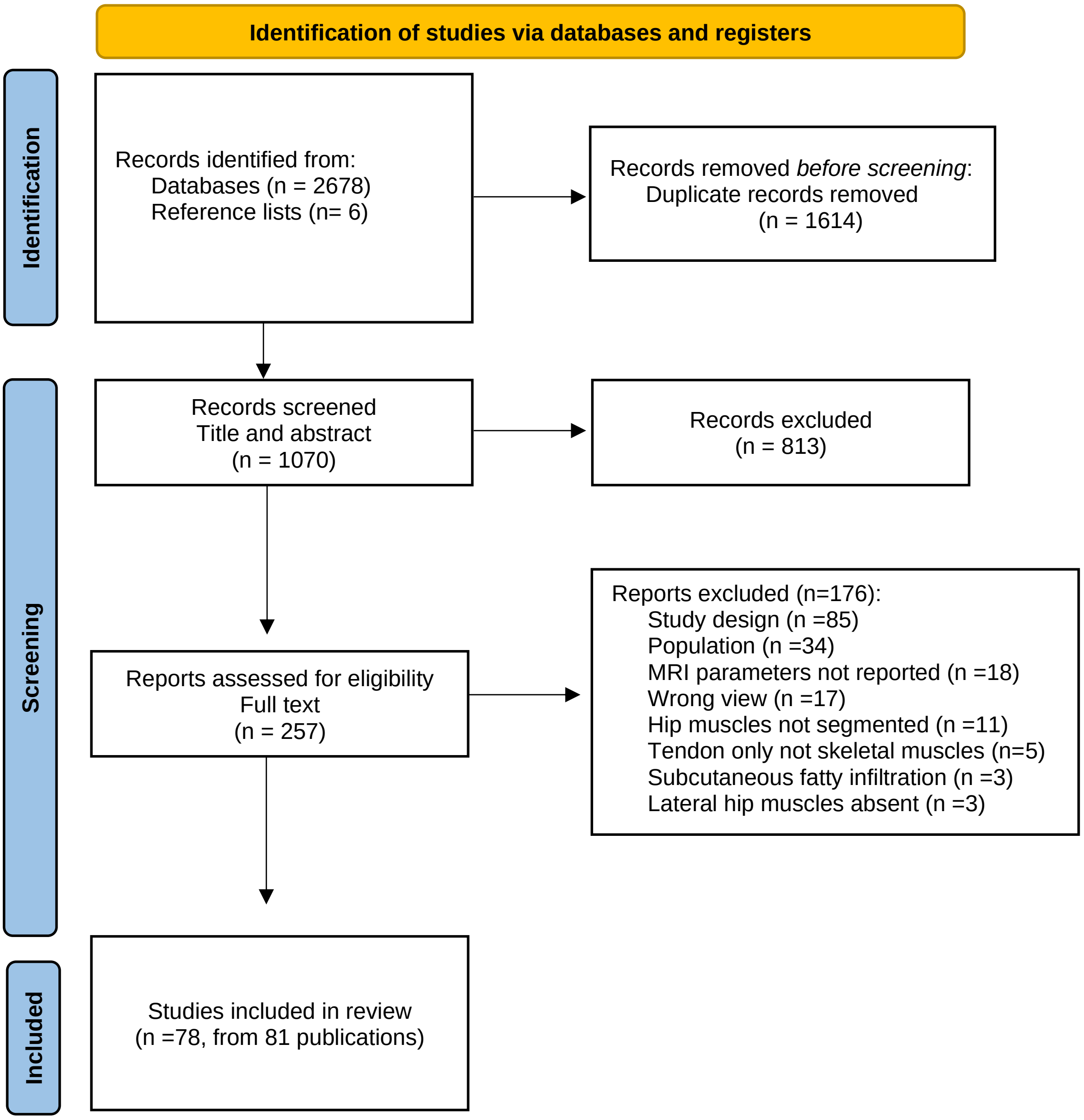

Supplement: Supplementary file 3 — Additional file 3. Preferred Reporting Items for Systematic reviews and Meta-Analyses extension for Scoping Reviews (PRISMA-ScR) (34) flow diagram. [file 12891_2022_5439_MOESM3_ESM.pdf]
